# *lncTCF7* is a negative prognostic factor, and knockdown of *lncTCF7* inhibits migration, proliferation and tumorigenicity in glioma

**DOI:** 10.1038/s41598-017-17340-y

**Published:** 2017-12-12

**Authors:** Xiao Gao, Xing Guo, Hao Xue, Wei Qiu, Xiaofan Guo, Jinsen Zhang, Mingyu Qian, Tong Li, Qinglin Liu, Jie Shen, Lin Deng, Gang Li

**Affiliations:** 1 Department of Neurosurgery, Qilu Hospital of Shandong University, Jinan, Shandong Province P.R. China; 2 Brian Science Research Institute, Shandong University, Jinan, Shandong Province P.R. China

## Abstract

Long noncoding RNAs (lncRNAs) have been shown to play critical roles in cancer. *lncTCF7* (gene symbol: WSPAR) has been reported to maintain stemness in hepatocellular carcinoma (HCC) stem cells. However, little is known about the role of *lncTCF7* in glioma. The aim of this study was to identify the role of *lncTCF7* in the pathogenesis of glioma. We analysed the relationship of *lncTCF7* expression with clinicopathological characteristics in glioma patients. Our results showed that *lncTCF7* expression was increased in glioma tissues compared with that in normal brain tissues (P < 0.001). Moreover, *lncTCF7* was significantly associated with WHO grade (I–II vs. III–IV; P = 0.006) and tumour size (<3 cm vs. T ≥ 3 cm; P = 0.025). Meanwhile, patients with high *lncTCF7* expression levels exhibited markedly worse overall survival prognoses (P < 0.01). Loss of function assays revealed that knockdown of *lncTCF7* significantly inhibited glioma cell migration, proliferation and tumorigenicity *in vitro* and *in vivo*. Furthermore, we found that hypoxia induced *lncTCF7* expression in an autocrine manner through IL-6 in glioma. In conclusion, *lncTCF7* may play a vital role in glioma progression and serves as a potential prognostic biomarker in glioma patients, providing new targets for glioma therapy.

## Introduction

Glioma is the most common primary tumour of the central nervous system and accounts for 81% of malignant brain tumours^1^. Despite extensive research over the past decades, the prognosis of glioblastoma (grade IV glioma) patients remains poor. However, emerging evidence has suggested that lncRNAs play an important role in cancer pathophysiology, providing new insight into the genetic and molecular mechanisms of cancer^2^. lncRNAs are defined as transcripts longer than 200 nucleotides that are 5′ capped and 3′ poly-adenylated; however, this class of transcripts has limited coding potential^3^. lncRNAs exert their functions via diverse mechanisms, including cotranscriptional regulation, modulation of gene expression, scaffolding of nuclear or cytoplasmic complexes, and pairing with other RNAs^4^. There have been several studies focusing on the role of lncRNAs, including *HOTAIR*, *MALAT1*, and *H19* ^5–9^, in glioma pathogenesis. However, no article has been published regarding *lncTCF7* in glioma.

*lncTCF7* can promote liver cancer stem cell (CSC) self-renewal and tumour propagation through activation of Wnt signalling by recruiting the SWI/SNF complex to the TCF7 promoter^10^. *lncTCF7* is highly expressed in HCC tissues and liver CSCs. In addition, *lncTCF7* depletion significantly reduced the expression of pluripotent transcription factors Sox2, Nanog, and Oct4, remarkably impairing the generation of the fraction of CD13 + CD133 + cells (CSCs), while *lncTCF7* overexpression enhances the tumorigenic capacity of liver CSCs. In this study, we aimed to explore the role of *lncTCF7* in glioma progression.

To explore the role of *lncTCF7* in glioma, we examined its expression level in 111 glioma tissues and 12 normal brain tissues. We found that the *lncTCF7* expression levels are higher in high-grade gliomas than in low-grade gliomas and normal brain tissues. Additionally, *lncTCF7* expression is associated with prognosis. Furthermore, the biological function of *lncTCF7* in proliferation, cell cycle, and migration was examined *in vitro*, and its function in tumorigenicity was also investigated in the nude mouse model.

## Results

### Increased expression of *lncTCF7* in Glioma

**Figure 1:**
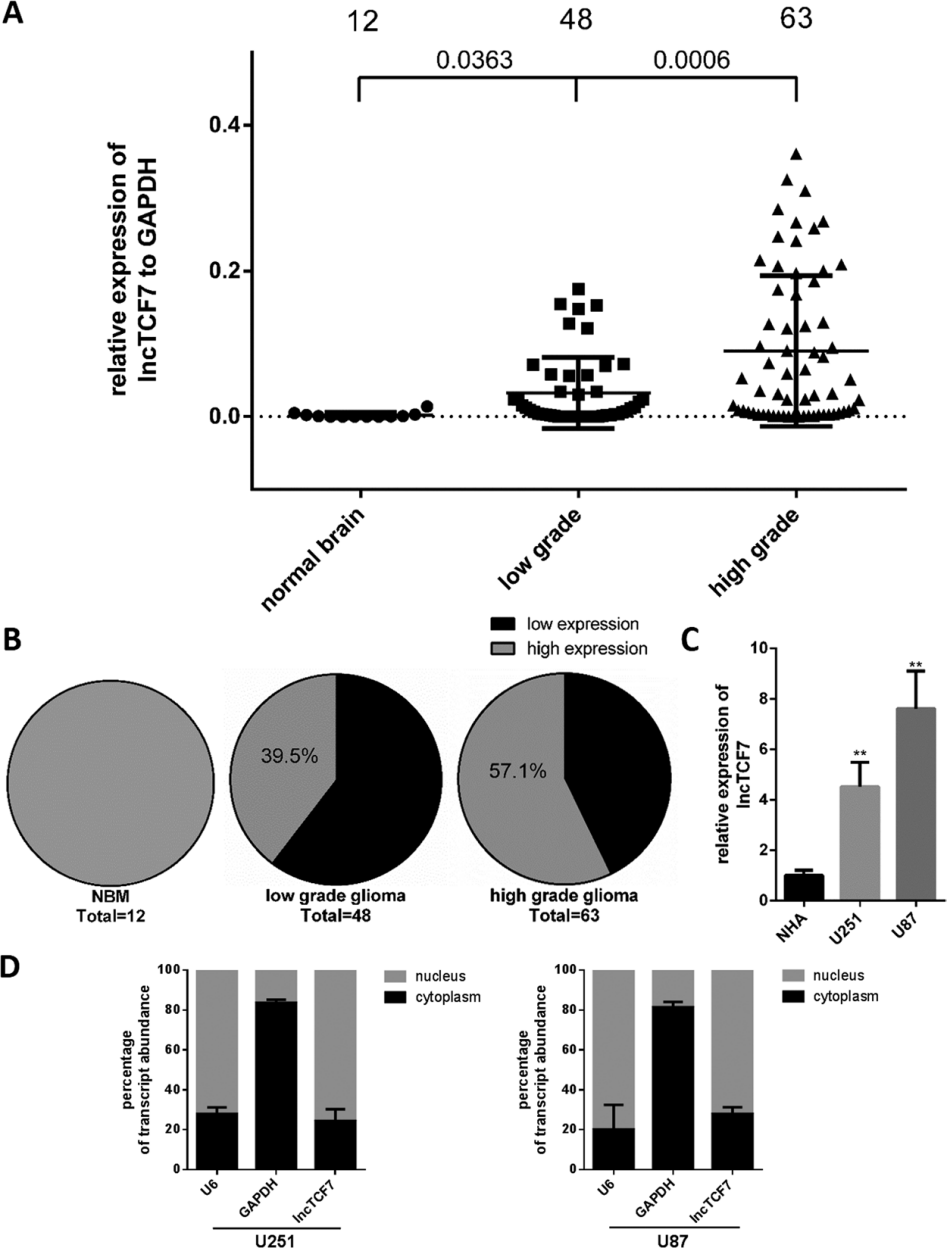
Quantitative determination of *lncTCF7* by qRT-PCR in glioma. (**A**) The relative expression level of *lncTCF7* in glioma tissues (n = 111) and normal brain tissues (n = 12) was measured by qRT-PCR. (**B**) Percentage of glioma patients with high or low *lncTCF7* expression level in different WHO grade groups. (**C**) The *lncTCF7* level was evaluated in glioma cells and normal human astrocytes (NHA). NBM, normal brain tissue. (**D**) Quantification of lncTCF7 in fractionated U251 and U87 cell lysates by qRT-PCR. U6 RNA served as a positive control for nuclear gene expression, and GAPDH served as a control for cytoplasmic gene expression.

To determine whether *lncTCF7* is involved in the development of glioma, we performed qRT-PCR to measure the expression levels of *lncTCF7* in 111 glioma tissues. Compared with 12 samples of normal brain tissue, *lncTCF7* expression in 111 glioma tissues was increased (P < 0.01, Fig. 1A). The expression of *lncTCF7* was higher in high-grade glioma than in low-grade glioma (P < 0.001, Fig. 1A). Additionally, the percentage of high *lncTCF7* expression was greater in high-grade glioma than in low-grade glioma (39.5% vs. 57.1%, respectively; Fig. 1B). Next, we examined *lncTCF7* expression in glioma cell lines (Fig. 1C) and discovered much higher expression of *lncTCF7* in U87 and U251 cell lines than in normal human astrocytes (NHA). Isolation of the nuclear and cytoplasmic fractions in U251 cells and U87 cells indicated that *lncTCF7* was mainly located in the nucleus (Fig. 1D). Thus, *lncTCF7* is upregulated in glioma and may serve as an oncogene in glioma development and progression.

### *lncTCF7* expression is correlated with clinicopathological features in glioma patients

We next analysed the correlation between the expression of *lncTCF7* and clinicopathological characteristics of glioma (Table 1). The clinicopathological characteristics of the 111 glioma patients presented in Table 1 showed that high expression of *lncTCF7* was significantly correlated with higher WHO grades (III/IV) and larger tumour size (å 3 cm). However, *lncTCF7* expression was not associated significantly with other parameters, such as age, gender and tumour location in glioma.

**Table 1:** Correlation between *lncTCF7* expression and clinicopathological characteristics in glioma.

Characteristics	n	High expression	Low expression	P
Age (years)				
≥45	64	34	30	0.13
<45	47	18	29	
Gender				
male	69	36	33	0.642
female	42	20	22	
WHO grade				
I-II	48	17	31	0.006
III-IV	63	39	24	
Tumour size (cm)				
≥3 cm	83	47	36	0.025
<3 cm	28	9	19	
Tumour location				
Supratentorial	95	48	47	0.969
Infratentorial	16	8	8	

The median expression level of *lncTCF7* was used as the cutoff.

### *lncTCF7* is an independent unfavourable prognostic factor in glioma

**Figure 2:**
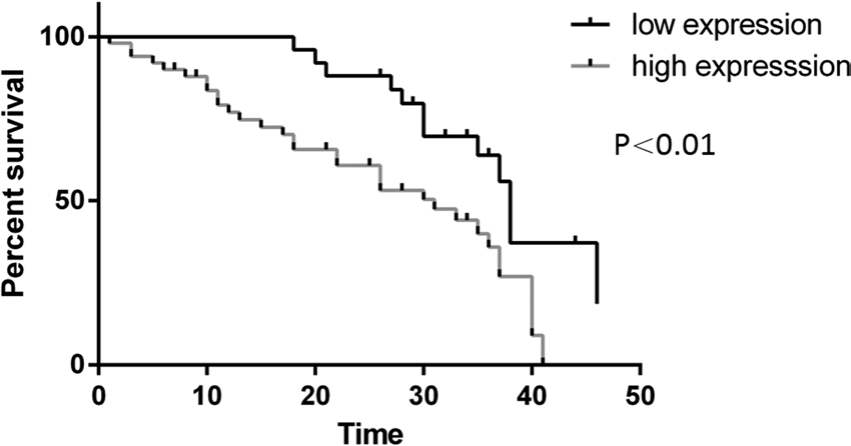
Increased *lncTCF7* expression predicts an unfavourable prognosis. The association between patient survival and *lncTCF7* expression was estimated using the Kaplan–Meier method and log-rank test (P < 0.01). Unit of time = months. Low expression (n = 55) vs. high expression (n = 56).

To explore the prognostic value of *lncTCF7* expression in glioma patients, we measured the association between *lncTCF7* expression and patient survival using Kaplan–Meier analysis. Patients with relatively higher expression of *lncTCF7* showed a worse prognosis than those with a lower expression (P < 0.01, Fig. 2). Thus, *lncTCF7* is a negative prognostic factor, and high *lncTCF7* expression is associated with poor overall survival (OS) in glioma. Furthermore, Cox regression analysis indicated that increased *lncTCF7* expression is an independent prognostic factor for glioma patients (Table 2).

**Table 2:** Univariate and multivariate analyses of overall survival rates by the Cox regression model.

Variables	Univariate analysis			Multivariate analysis		
	HR	95% CI	P	HR	95% CI	P
Age (years)						
<45 vs. ≥ 45	2.641	1.172–5.951	0.019	2.025	0.867–4.728	0.103
Gender						
Male vs. female	1.630	0.725–3.666	0.237			
WHO grade						
I-II vs. III-IV	2.470	1.098–5.554	0.029	1.503	0.631–3.581	0.358
Tumour size (cm)						
<3 vs.≥3	1.200	0.453–3.182	0.714			
Tumour location						
Supratentorial vs. infratentorial	1.694	0.513–5.593	0.387			
*lncTCF7* expression						
low vs. high	3.211	1.377–7.489	0.007	2.593	1.071–6.280	0.035

HR, hazard ratio; CI, confidence interval.

### Knockdown of *lncTCF7* inhibits glioma cell migration

**Figure 3:**
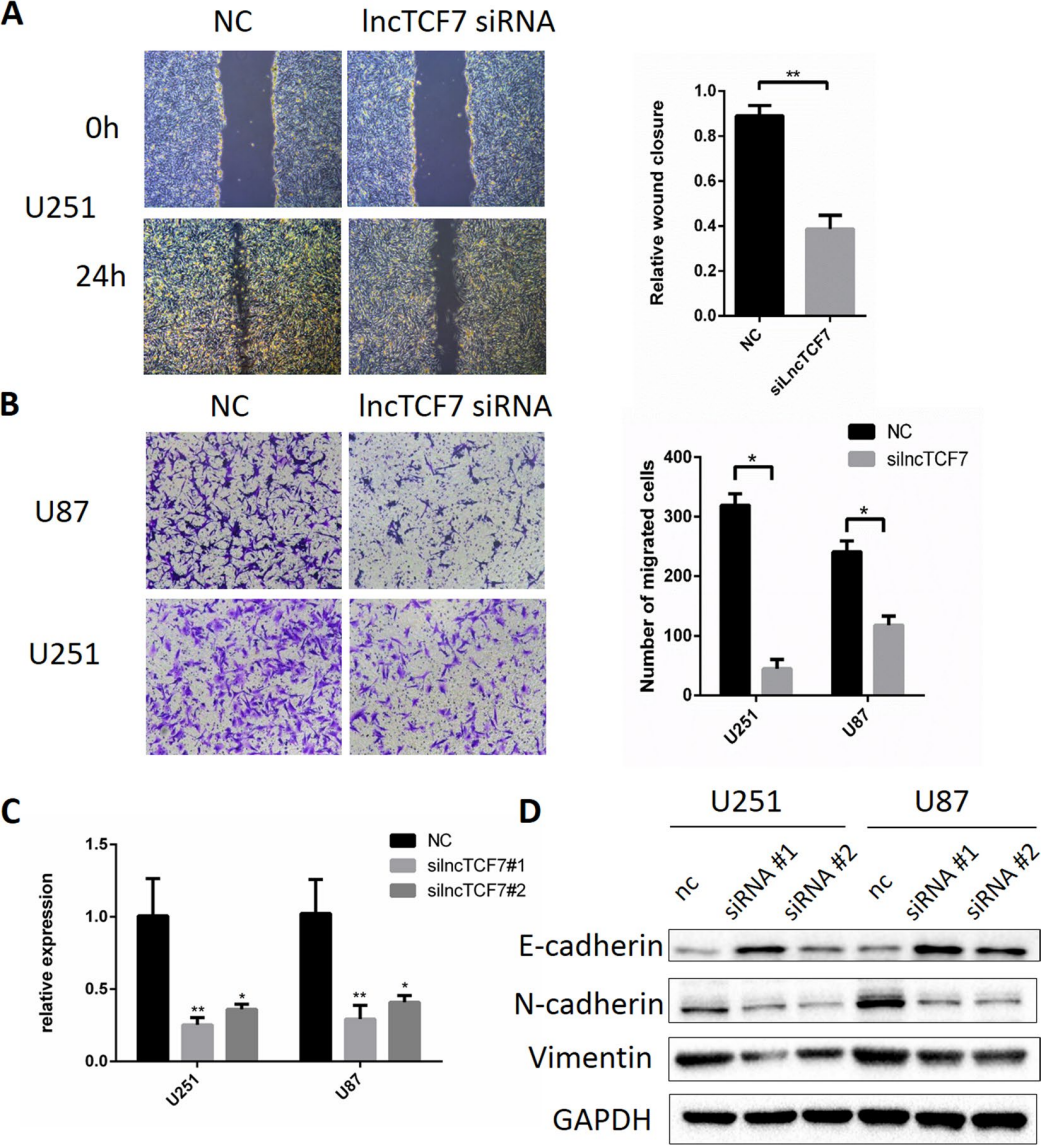
Knockdown of *lncTCF7* inhibits the migration ability of glioma cells. (**A**) Wound healing assay and (**B**) transwell assay showing U251 and U87 cells with knockdown of *lncTCF7*, compared with negative controls. (**C**) The knockdown effects of *lncTCF7* were measured by qRT-PCR in glioma cells transfected with siRNA#1, siRNA #2 or its negative controls. (**D**) Effects of *lncTCF7* knockdown by siRNA on the protein expression of EMT markers in U251 and U87 cells.

To assess the potential functional role of *lncTCF7*, two siRNAs were synthesized, and the efficiency was measured in glioma cell lines by qRT-PCR (Fig. 3C). Wound healing assays and transwell assays were employed to detect the effects of si-*lncTCF7* on the migratory ability of glioma cells (Fig. 3A,B). As shown in these two assays, knockdown of *lncTCF7* reduced cellular migration in glioma cells dramatically, indicating that *lncTCF7* is important for glioma cell migration. Similar results of wound healing assays were observed in U251 cells. Considering the tight relationship between the epithelial-mesenchymal transition (EMT) and cellular migration, several EMT-related protein markers were determined by western blotting (Fig. 3D). Knockdown of *lncTCF7* significantly decreased the expression of N-cadherin and Vimentin, as well as increased the expression of E-cadherin, which mediates stable intercellular adhesions. These results suggest that knockdown of *lncTCF7* suppressed the mesenchymal phenotype and inhibited migration through hindering EMT in glioma cells. Therefore, *lncTCF7* may promote glioma progression by enhancing cell migration.

### Knockdown of *lncTCF7* inhibits proliferation and induces G1 arrest in glioma cells *in vitro*

**Figure 4:**
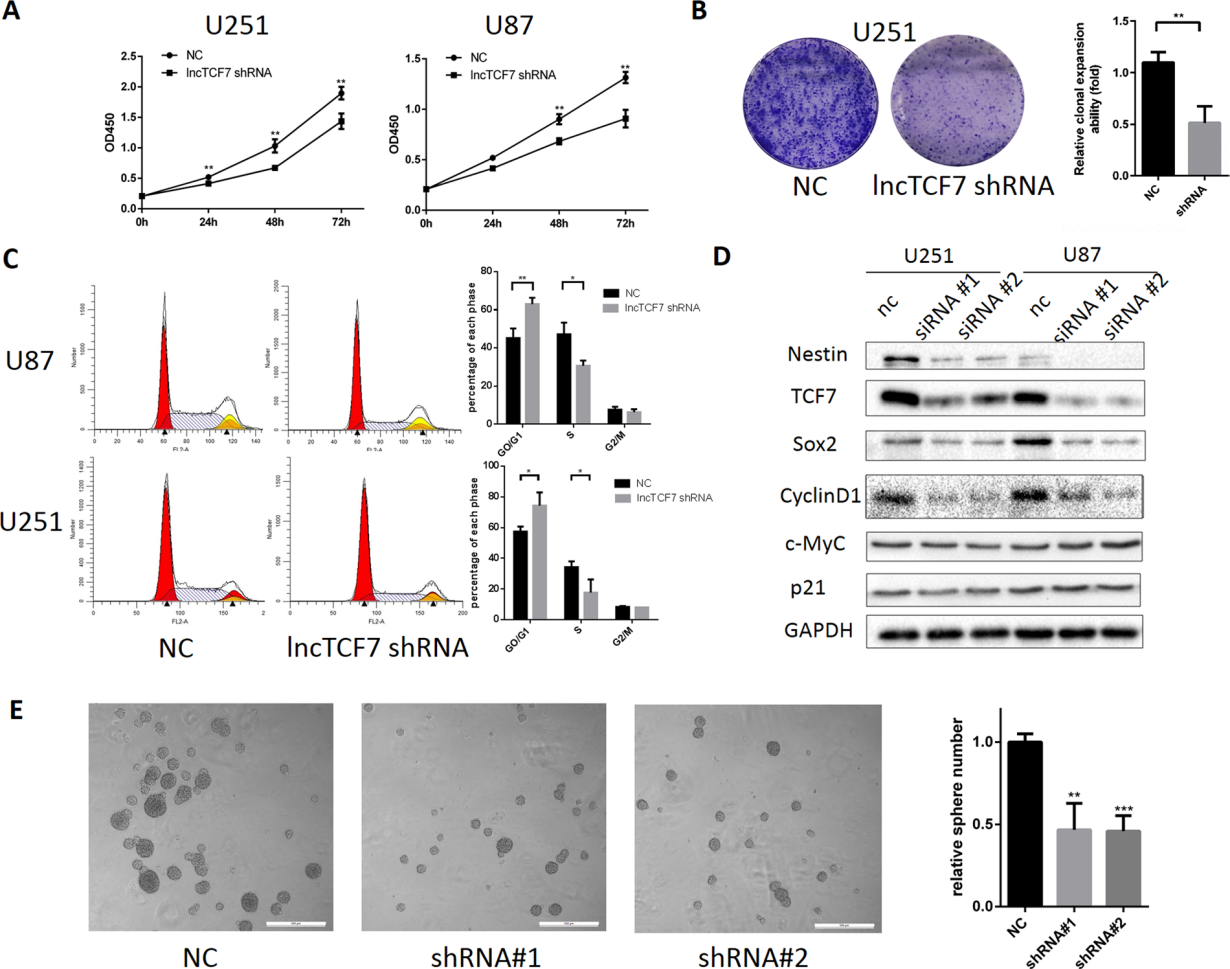
Knockdown of *lncTCF7* inhibits glioma cell growth and results in the G0/G1 cell cycle G1 arrest. U87 and U251 cells were transfected with *lncTCF7* shRNA, and the proliferation were tested by CCK-8 assay (**A**) and colony formation assay (**B**,**C**). U87 and U251 cells were transiently transfected with *lncTCF7* shRNAs or NC shRNAs for 48 h, and the cell cycle profiles were examined by flow cytometry. (**D**) Knockdown of *lncTCF7* inhibited TCF7, Sox2 and CyclinD1 expression through hindering Wnt signalling. Full-length images of cropped gels from Figs 3D and 4D are shown in Supplementary Information file. (**E**) Knockdown of *lncTCF7* inhibited sphere formation in glioma stem cells. The right panel presents the statistical results as mean as the means ± SD. Scale bar, 500 μm.

We further examined whether knockdown of *lncTCF7* affects the proliferation of glioma cells. U251 and U87 cells were transfected with shRNA#1, and CCK-8 assays (Fig. 4A) were performed to measure the viability at each time point (0 h, 24 h, 48 h, 72 h). Knockdown of *lncTCF7* reduced not only the CCK-8 absorbance but also the colony number in colony formation assays (Fig. 4B). Given that cell cycle progression is associated with proliferation, we next used propidium iodide (PI) staining to analyse the effect of *lncTCF7* knockdown on glioma cell proliferation by flow cytometry (Fig. 4C). Compared with the respective control groups, *lncTCF7* knockdown groups showed a decrease in the percentage of cells in S phase and a marked accumulation in the percentage of cells in the G0/G1 phase in U87 and U251 cells. Meanwhile, the sphere formation capacity of glioma stem cells (GSCs) was also impaired by *lncTCF* knockdown (Fig. 4E), suggesting that *lncTCF7* may have a direct impact on the stemness and tumorigenicity of glioma.

Furthermore, several cell cycle-related proteins were detected by western blotting (Fig. 4D), showing that CyclinD1, one of the direct target genes of Wnt/b-catenin signalling and responsible for G1/S transition^11–13^, was subsequently downregulated after *lncTCF7* shRNA transfection. However, c-Myc expression did not decrease. In the meantime, p21, the cyclin-dependent kinase inhibitor which is downregulated by c-Myc, was not changed. These results implied that knockdown of *lncTCF7* induced G1 arrest through the downregulation of CyclinD1 rather than c-Myc/p21 by suppressing the Wnt/b-catenin pathway. Moreover, Sox2, another target gene of Wnt/b-catenin signalling, was downregulated by the knockdown of *lncTCF7*, indicating that the proliferation reduction resulted from cylinD1 downregulation-mediated G1 arrest, as well as Sox2 downregulation-mediated self-renewal and tumorigenicity impairment. Nestin is a protein marker for neural stem cells and glioma stem cells^14,15^ and is associated with poor clinicopathological features and prognosis in glioma patients^16^. Knockdown of *lncTCF7* decreased the expression of Nestin, indicating *lncTCF7* is important for glioma cells to maintain stemness.

### Knockdown of *lncTCF7* reduces glioma growth *in vivo*

**Figure 5:**
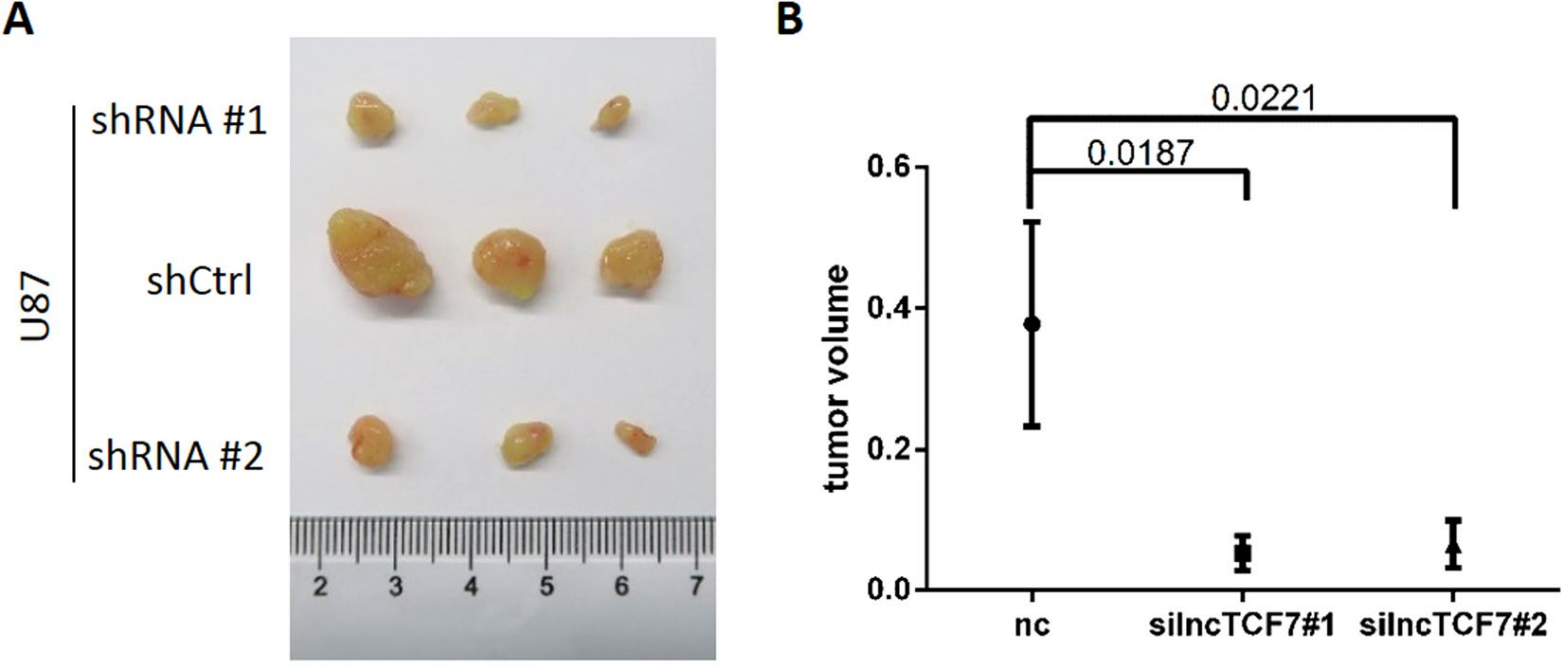
Knockdown of *lncTCF7* reduces glioma growth *in vivo*. (**A**) The tumours 28 days after U87MG cells with shCtrl or shRNA targeted *lncTCF7* (sh*lncTCF7*) were subcutaneously injected into the flanks of nude mouse. (**B**) Tumour volume comparison.

We further validated the effects of *lncTCF7* downregulation *in vivo* by injecting U87 glioma cells into nude mice. As shown in Fig. 5A, down-regulation of *lncTCF7* expression significantly reduced the tumour volume compared with that in the control group.

### Hypoxia-induced IL-6 secretion increases *lncTCF7* expression

**Figure 6:**
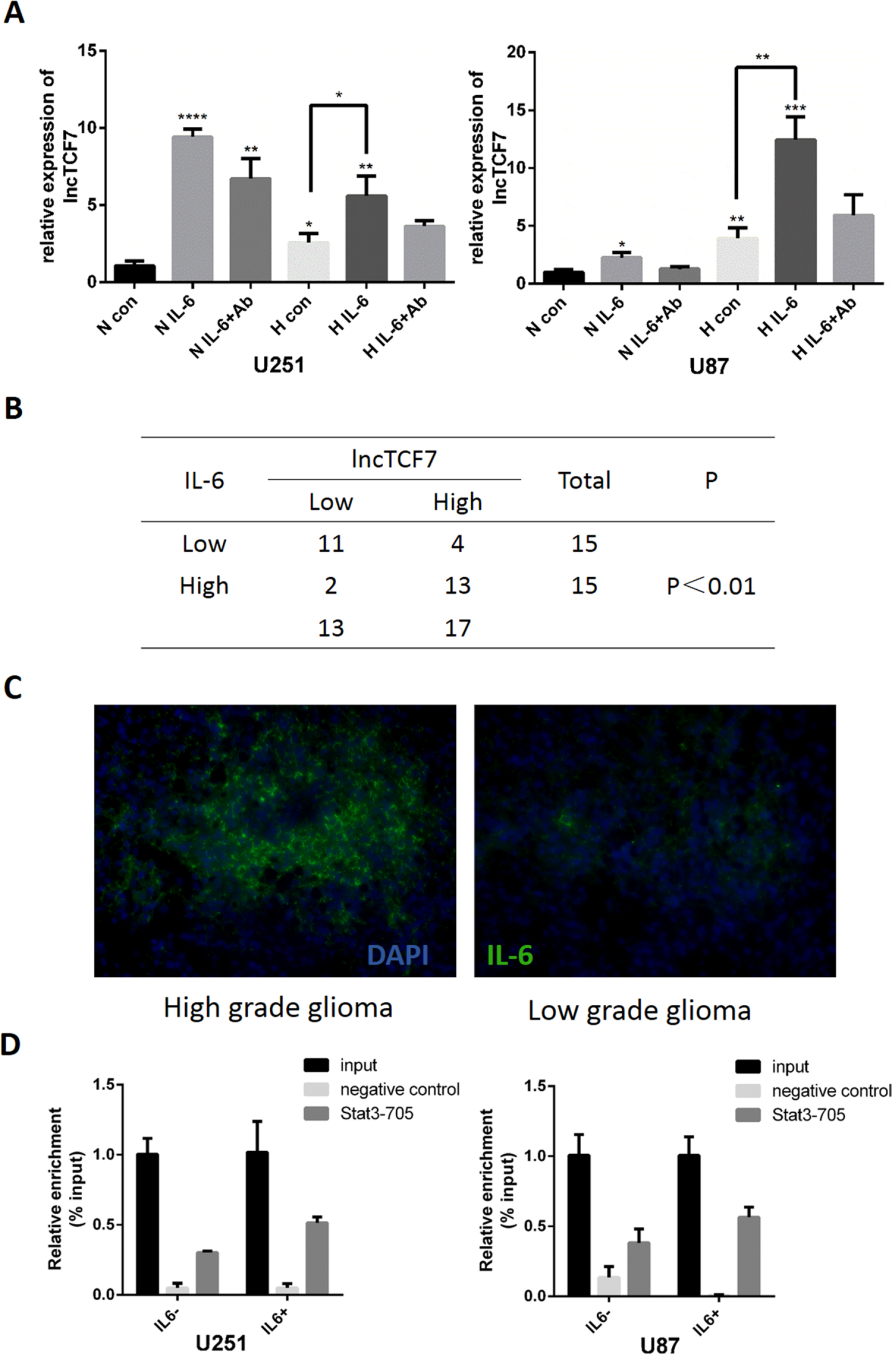
Hypoxia-induced IL-6 secretion increases *lncTCF7* expression. (**A**) Hypoxia increased *lncTCF7* expression significantly. Under hypoxia (H) and normoxia (N) conditions, respectively, exogenous IL-6 (20 ng/ml) increased *lncTCF7* expression in U87 and U251 cells, while IL6 antibody (Ab, 1 μg/ml) did not inhibit *lncTCF7* expression completely. (**B**) Association between IL-6 and *lncTCF7*. IL-6 and *lncTCF7* expression in 30 glioma tissue specimens was analysed by Fisher’s exact test. (**C**) Representative images of high-grade and low-grade glioma showed immunofluorescence staining of IL-6. (**D**) Binding of Stat3 onto the *lncTCF7* promoter regions was examined via ChIP assay in glioma cells U251 and U87MG treated with or without IL-6 (20 ng/ml).

Next, we investigated the mechanisms underlying the upregulation of *lncTCF7* in glioma. Hypoxia is an important factor that promotes and maintains the stemness of CSCs and other stem cells^17,18^. Within GBM, distinct cancer stem cell niches are often located in the perivascular and hypoxic regions^19^. We detected *lncTCF7* expression after hypoxia (1% O_2_) treatment in both U251 and U87 cell lines. Compared with the normoxia group, *lncTCF7* expression was increased under the hypoxia condition in both cell lines (P < 0.01, Fig. 6A). Hypoxia-inducible factors (HIFs) are the main modulators during hypoxia. However, we failed to identify a HRE (HIF-response element) site on the promoter of *lncTCF7*.

While hypoxia can also influence the downstream pathways through modulating inflammatory cytokine secretion^20^, interleukin 6 (IL6) was reported to induce *lncTCF7* expression. IL-6 transcriptionally activated the expression of *lncTCF7* in HCC cells by activating STAT3^21^. In both normoxia and hypoxia, IL-6 induced *lncTCF7* expression obviously (P < 0.01, Fig. 6A). Chromatin immunoprecipitation (ChIP) assay was performed to examine the interaction between the STAT3 and *lncTCF7* promoter. Our results showed that the binding of STAT3 to the *lncTCF7* promoter region was similar in both glioma cell lines, especially with the IL-6 (20 ng/ml) stimulus (Fig. 6D).

In addition, the IL-6-*lncTCF7* axis was also validated in patient samples. As shown in Fig. 6B,C, a higher expression level of *lncTCF7* was accompanied by higher IL-6 expression, and the association analysis revealed that *lncTCF7* expression was associated with IL-6 expression (P < 0.01, Fig. 6B). However, the IL-6-neutralizing antibody was not sufficient to block *lncTCF7* expression (Fig. 6A), indicating that other compensate pathways may be involved in *lncTCF7* regulation and that anti-IL-6 therapy alone may promote resistance.

**Figure 7:**
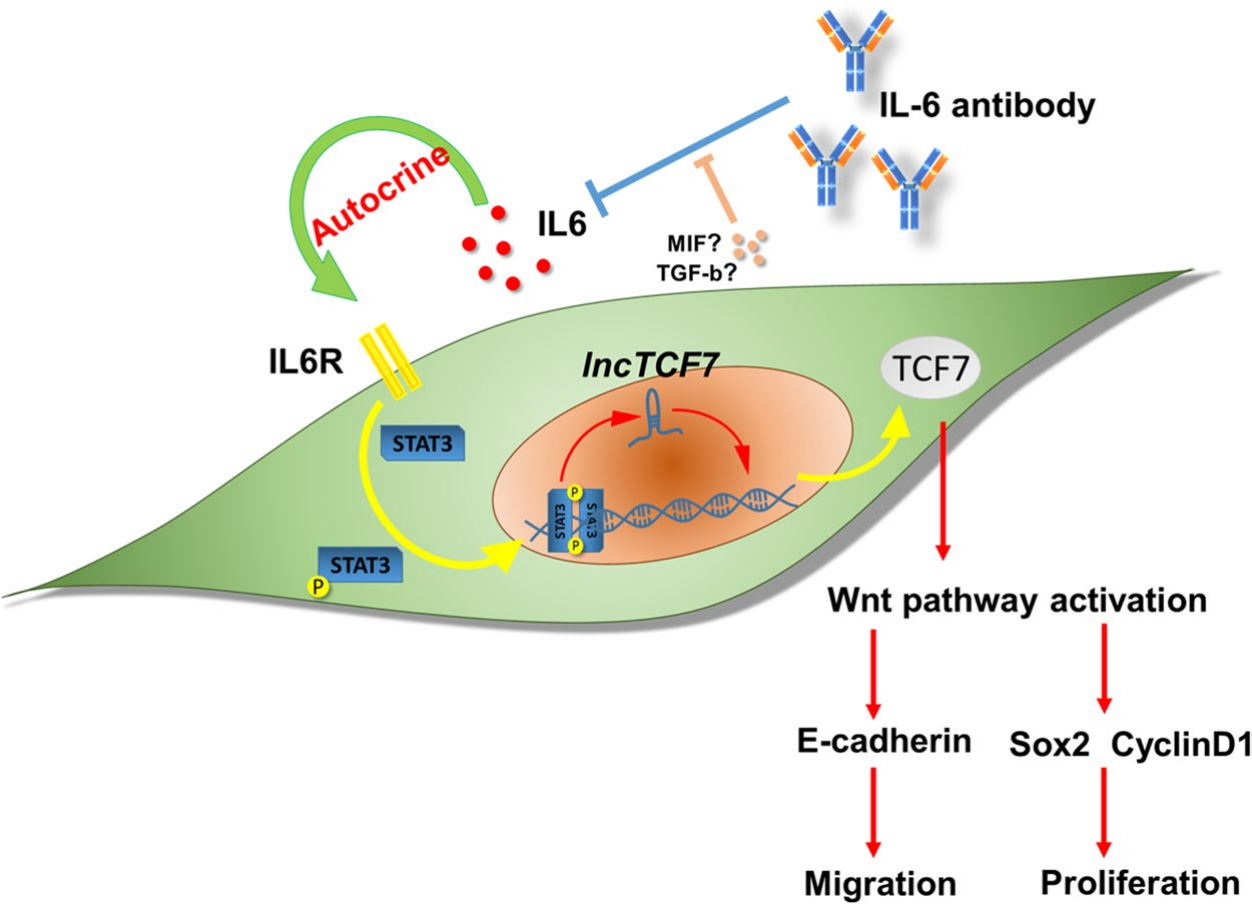
Schematic diagram of the hypoxia/IL-6/*lncTCF7*/TCF7/Wnt signalling axis in glioma. Hypoxia-induced IL-6 secretion increases *lncTCF7* expression. *LncTCF7* activates the Wnt signalling pathway through TCF7 expression to promote the migration and proliferation of glioma cells.

All together, these data showed that hypoxia-induced IL-6 secretion increases *lncTCF7* expression, which is important for the migration and proliferation of glioma cells (Fig. 7).

## Discussion

Advances in human transcriptome analysis has revealed that less than 2% of the transcriptional output encodes proteins, and the remaining 98% serve as different classes of non-coding RNAs^22^. lncRNA, as a new class of non-coding RNAs, has attracted the increased attention of researchers.

Recent studies have investigated the regulatory roles of *lncTCF7* in liver and lung cancer progression. Yanying Wang *et al*. first found *lncTCF7* as a key regulator of liver cancer stem cell self-renewal and tumour propagation through interfering with Wnt signalling^10^. Jun Wu *et al*. found that STAT3, which is activated by exogenous IL-6, can directly bind to the promoter regions of *lncTCF7* to activate its transcription. They provided evidence that this aberrant IL-6/STAT3/ *lncTCF7* signalling axis promotes HCC aggressiveness through EMT induction^21^. The role of *lncTCF7* was also investigated in lung cancer. *lncTCF7* was proven to promote the invasiveness and self-renewal of NSCLC cells^23^.

The Wnt signalling pathway controls a myriad of biological processes throughout development and the adult life of all animals^24^. Cell proliferation and migration are two aspects in which Wnt signalling plays a vital role.

Increased β-catenin can initiate the transcriptional activation of proteins such as CyclinD1 and c-Myc, which control the G1-to-S phase transition in the cell cycle^25^. In addition, Wnt signalling is an inducer of EMT and can promote cell migration by interfering with cell adhesion^26^. Therefore, it is not difficult to understand that Wnt signalling underlies a wide range of diseases in humans, especially cancer. Increased expression of Wnt ligand proteins were observed in the development of glioblastoma, oesophageal cancer and ovarian cancer^27^. In glioma, the Wnt signalling pathway is also one of the key signalling pathways for development and progression^28,29^. Meanwhile, recent studies have revealed that many lncRNAs could regulate the Wnt/b-catenin signalling pathway in several types of cancer. Yun Cui’s study showed that lncRNA SNHG1 promotes lung cancer tumorigenesis and progression via the miR-101-3p/SOX9/Wnt/b-catenin axis^30^. Zheying Zhang *et al*. revealed that lncRNA CASC11 promotes colorectal cancer cell proliferation and metastasis by associating with hnRNP-K and activating WNT/β-catenin signalling^31^. Hongying Liu *et al*. demonstrated that the knockdown of lncRNA UCA1 inhibits Wnt/b-catenin signalling and increases tamoxifen sensitivity in breast cancer^32^.

In the present study, we first found that *lncTCF7* was overexpressed in glioma tissues and U251 and U87 cell lines, and its expression was positively correlated with advanced tumour stage and tumour size. Meanwhile, increased *lncTCF7* expression predicted an unfavourable prognosis in glioma patients. Furthermore, the loss of function assay revealed that knockdown of *lncTCF7* significantly inhibited glioma cell migration, proliferation and tumorigenicity through upregulating E-cadherin or suppressing TCF7/Sox2 and CyclinD1 expression.

Hypoxia is one of the important characteristics of solid tumours, including glioma, and is associated with worse patient survival and therapeutic resistance. Recently, dozens of lncRNAs have been reported to be dysregulated in hypoxia and exert diverse functions in tumour biology^33^. IL6 is a potent cytokine that regulates cell growth and differentiation and plays an important role in the immune response^34,35^. Increased or deregulated expression of IL6 significantly contributes to the pathogenesis of various human diseases, including tumours. Consistent with Jun Wu’s study^21^, we found that IL-6 induced *lncTCF7* expression in glioma cells (Fig. 6). Based on our previous study^20^, we investigated whether the IL-6 blocking antibody can serve as a potential therapy targeting the IL-6/*lncTCF7*/Wnt pathway. However, the IL-6 antibody did not reduce *lncTCF7* expression to basal levels (Fig. 6A), indicating the presence of another compensatory pathway involving other pro-inflammatory cytokines, such as macrophage migration inhibitory factor (MIF) and transforming growth factor beta (TGF-β). Further research into the regulation of *lncTCF7* may provide a solution for anti-IL-6 therapy resistance.

In summary, we demonstrated that *lncTCF7* is a negative prognostic factor in glioma and that down-regulation of lncTC7 expression inhibits glioma cell migration, proliferation and tumourigenicity potential by suppressing the Wnt signalling pathway. Furthermore, hypoxia-induced IL-6 secretion increases *lncTCF7* expression. Therefore, the hypoxia/IL-6/*lncTCF7* axis may be a potential target for glioma therapy.

## Materials and Methods

### Patients samples

This study was performed in accordance with relevant guidelines and was approved by the Ethics Committee of Qilu Hospital. Informed consent for both study participation and publication was obtained from all patients. One hundred eleven glioma tissues were obtained from patients with glioma who underwent initial surgery at Qilu Hospital. The specimens were snap frozen in liquid nitrogen for real-time PCR. Overall survival (OS) was defined as the interval between the dates of surgery and death, and 74 patients were followed up successfully.

### RNA extraction and quantitative real-time PCR

Total RNA was extracted using TRIzol (Invitrogen) according to the manufacturer’s protocol. Total RNA was reverse transcribed into cDNA using the ReverTraAce qRT-PCR kit (FSQ-101; Toyobo). Real-time PCR was performed using the Lightcycler 480 SYBR GREEN I MASTER (Roche). The primers used for *lncTCF7* and GAPDH were as follows: GAPDH-F: 5′-GCACCGTCAAGGCTGAGAAC-3′, R: 5′-TGGTGAAGACGCCAGTGGA-3′; *lncTCF7*-F: 5′-AGGAGTCCTTGGACCTGAGC-3′, R: 5′-AGTGGCTGGCATATAACCAACA-3′. All of the data for each sample were collected in triplicate. Standard curves were generated, and the relative amount of *lncTCF7* was normalized to the amount of GAPDH (2^−ΔCt^).

### *lncTCF7* knockdown and transfection

Both siRNA and shRNA were used for *lncTCF7* knockdown. We employed siRNA in migration assay and western blotting, while used shRNA to get a sustained knockdown effect in proliferation-related assay. The effective shRNAs were as follows^10^: *lncTCF7* 1#:5′-AGCCAACATTGTTGGTTAT-3′, 2# 5′-CACCTAGGTGCTCACTGAA-3′; the corresponding siRNAs were designed by Gene Pharma (Shanghai, China): *lncTCF7* 1#:5′-AGCCAACAUUGUUGGUUAUTT-3′, 2#:5′-CACCUAGGUGCUCACUGAATT-3′; the control siRNA (negative control, NC) was as follows: 5′-UUCUCCGAACGUGUCACGUTT-3′.

For siRNAs transfection, cells were seeded into 6-well plates at 10^6^ cells per well. 24 h later, cells were transfected with siRNAs or the negative control sequences using Lipofectamine 2000 Transfection Reagent (11668–019, Life Technologies, CA, USA) according to the manufacturer’s instructions. The medium was replaced 6 h after the transfection. Transfected cells were harvested for other experiments after another 36 h.

For knockdown with shRNAs, shRNAs were constructed into the pcDNA3.1 plasmid. Cells were seeded in a 6-well plate at a concentration of 1.5 × 10^6^ cells per well and allowed overnight growth to reach 70–80% confluency. Cells were then transfected with the mixture of 1 μg of plasmid DNA and 8 μl of Lipofectamine 2000 in 1.5 ml of serum-free medium. Fetal bovine serum (200 μl/well) was added at 6 h post-transfection. At 24 h post-transfection, the medium was replaced by complete medium and cultured up to 48 h after transfection for proliferation-related assay.

For xenograft assay, U87 cells transfected with pcDNA3.1- *lncTCF7* shRNA were selected with G418. At 48 h post-transfection, medium containing G418 was added and the medium was replaced every 2 days. After 3 passages, U87 cells with stable knockdown of *lncTCF7* were obtained for xenograft assay.

### Cell proliferation and colony formation assay

Cell proliferation was detected by the Cell Counting Kit-8 (CCK-8). 48 h after transfection with shRNA, U251 or U87 (2.0 × 10^3^) cells were plated into 96-well plates and were cultured for 1, 2, 3 or 4 days. Next, 10 μl of CCK-8 (Dojindo, Japan) in 100 μl of culture medium was added to each well and was incubated for a further 1 h at 37 °C. The absorbance was measured at a wavelength of 450 nm. For the colony formation assay, 2000 cells per well were seeded into a six-well plate after transfection. Approximately 10 days later, the colonies were fixed in 4% methanol for 30 min and were stained with 1% crystal violet overnight, and then the number of colonies was counted.

### Cell cycle analysis

For cell cycle analysis, after the transfected cells described above were seeded on six-well plates for 48 h, the cells were collected by centrifugation and then were fixed with 70% ethanol at 4 °C overnight. Finally, the cellular DNA content was analysed by flow cytometry after propidium iodide (PI) staining (BD C6, BD Biosciences) using ModFit LT v2.0 software.

### Chromatin immunoprecipitation (ChIP) assay

ChIP assays were performed using the EZ-Magna ChIP kit (Millipore, Temecula, CA, USA) according to the manufacturer’s instructions. In brief, U251 or U87MG cells were fixed in 1% formaldehyde for 10 min at room temperature and were sonicated to prepare chromatin fragments. The genomic DNA fragments were immunoprecipitated with antibodies against phosphor-Stat3(TyR705) (Cell Signaling Technology) or normal rabbit IgG at 4 °C overnight. After crosslinking reversal and DNA clean-up, purified precipitated DNA was analysed by quantitative real-time PCR using primers targeting the *lncTCF7* promoter region: 5′-AGCCAGACAGAAGAGTGGA-3′and 5′-TGGGATGGGGATGTCAGAAC-3′.

### Sphere formation assay

Glioma stem cells were cultured from the primary cells of one glioma patient sample in serum-free Neurobasal-A medium (Invitrogen) containing 1 × penicillin G and streptomycin supplemented with 20 ng/ml epithelial growth factor (Peprotech), 10 ng/ml fibroblast growth factor-2 (Peprotech), and B27 (Invitrogen) at 37 °C in 5% CO_2_. Additionally, these cells were identified as glioma stem cells by immunofluorescence with antibodies against Nestin and Sox2. After digestion into single cells, glioma stem cells with the scrambled sequence or shRNA were seeded onto six-well plates. After one week, the numbers of spheres were counted under a stereomicroscope.

### Xenograft growth in nude mice

Animal studies were performed in accordance with the national guidelines of the Institutional Animal Care and Use Committee. For subcutaneous injection models, 10^6^ U87 cells were implanted into mice (male BALB/c nude mice), aged 4 weeks, at the posterior dorsal flank region (n = 3 per group). The tumours were measured every other day. All experimental procedures were approved by the ethical committee of Qilu Hospital. The mice were maintained under standard conditions according to the institutional guidelines for animal care.

### Statistical analysis

Data analyses were conducted using SPSS 20.0 and GraphPad Prism 6. Data are presented as the means ± SD. Chi-square test was used to evaluate the relationship between the *lncTCF7* expression profiles and clinicopathological features. Comparisons between groups were analysed using Student’s test or ANOVA. Kaplan–Meier analysis was used to evaluate the probability of patient survival, and distributions were evaluated using the log-rank test. Cox regression models were used to calculate hazard ratios and identify factors affecting survival. For all statistical analyses, P < 0.05 was considered statistically significant.

### Data availability

The data that support the findings of this study are available from the corresponding author upon reasonable request.

## Electronic supplementary material

Supplementary information
