# Supplementary material for: lncTCF7 is a negative prognostic factor, and knockdown of lncTCF7 inhibits migration, proliferation and tumorigenicity in glioma
Source: Sci Rep. 2017 Dec 12;7:17456. doi: 10.1038/s41598-017-17340-y (PMC5727168; doi:10.1038/s41598-017-17340-y)

LncTCF7 is a negative prognostic factor and knockdown of *lncTCF7* inhibits migration, proliferation and tumorigenicity in glioma

Xiao Gao[1], Xing Guo[1], Hao Xue[1], Wei Qiu[1], Xiaofan Guo[1], Jinsen Zhang[1], Mingyu Qian[1], Tong Li[1], Qinglin Liu[1], Jie Shen[1], Lin Deng[1], Gang Li[1,2]

[1]Department of Neurosurgery, Qilu Hospital of Shandong University, Jinan, Shandong Province, P.R. China

[2]Brian Science Research Institute, Shandong University, Jinan, Shandong Province, P.R. China

Correspondence to:
Gang Li
Department of Neurosurgery, Qilu Hospital of Shandong University
107 West Wen Hua Road
Jinan, China.
Tel & Fax: +86-531-82166615.
E-mail: ligangqiluhospital@163.com

Images of full-length gels

Fig 3. D

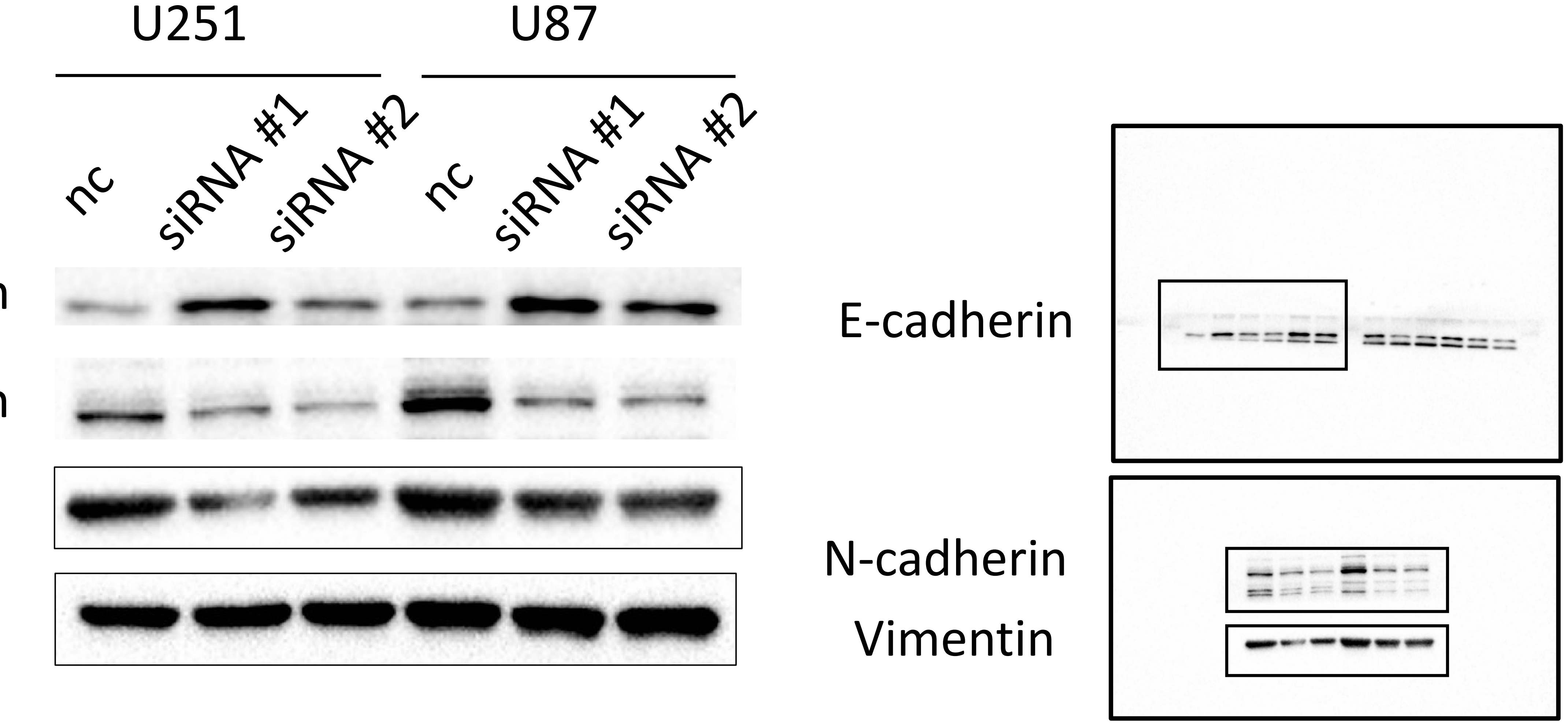

Fig 4. D

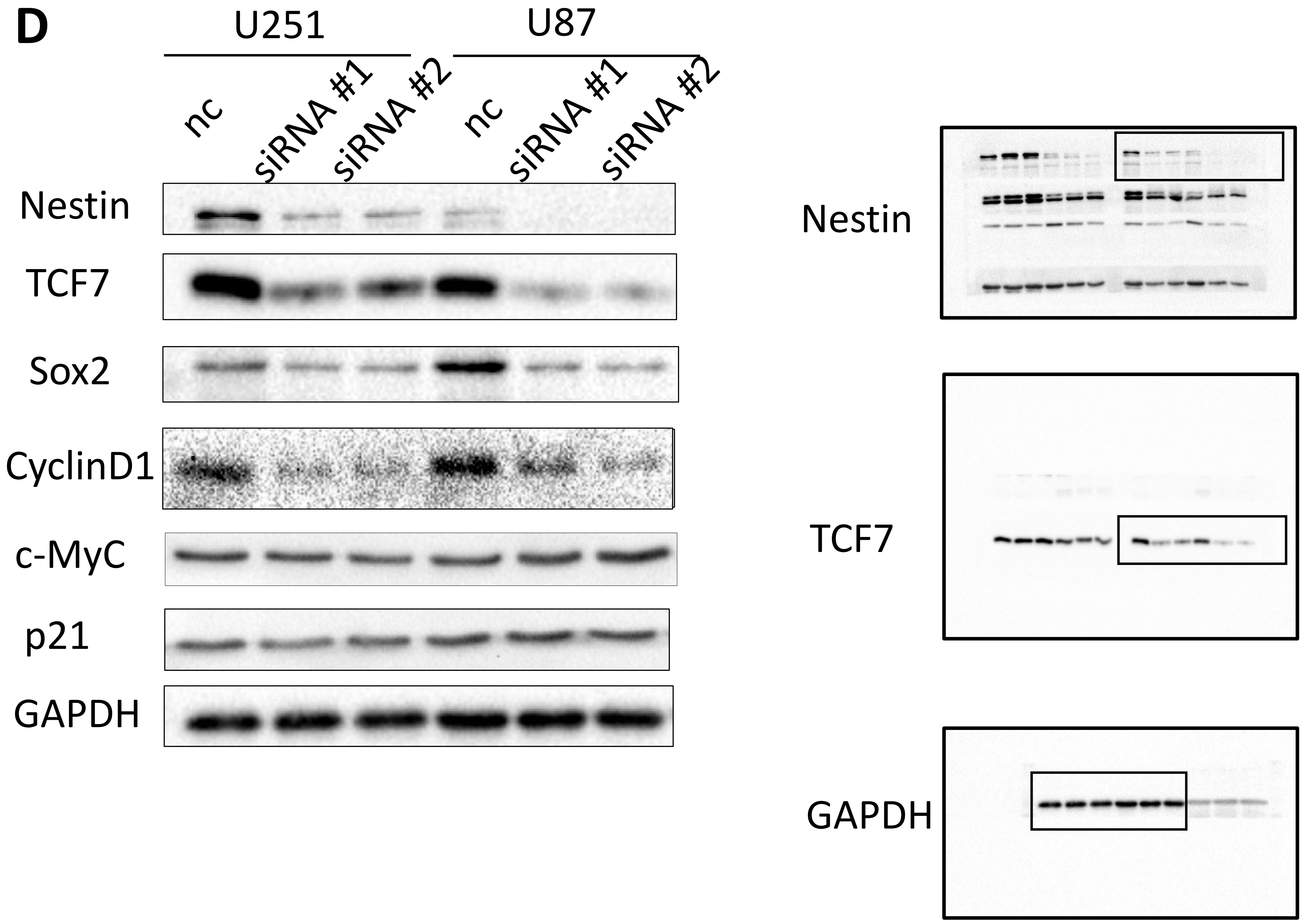

Fig 3. D

E-cadherin

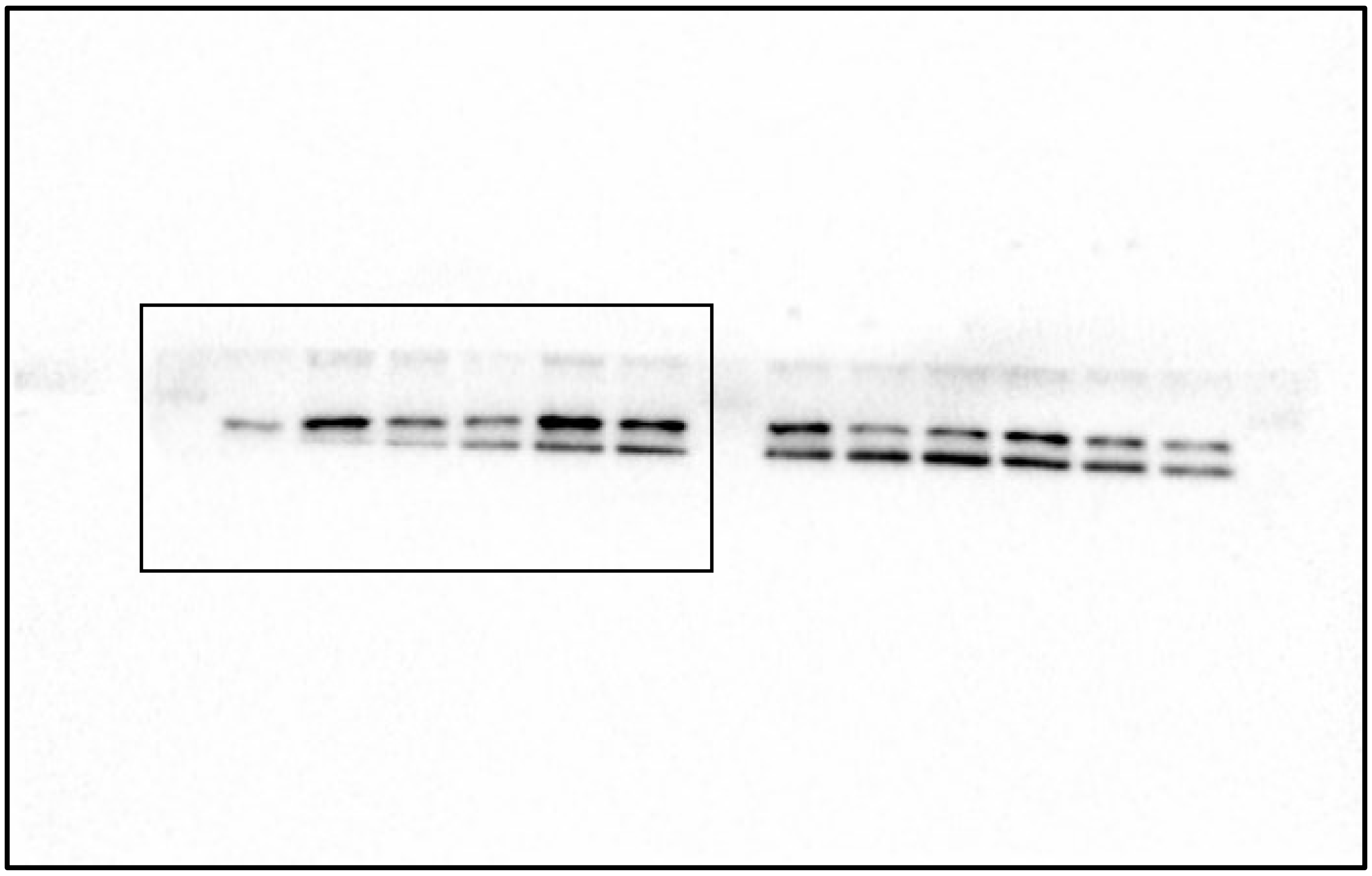

N-cadherin

Vimentin

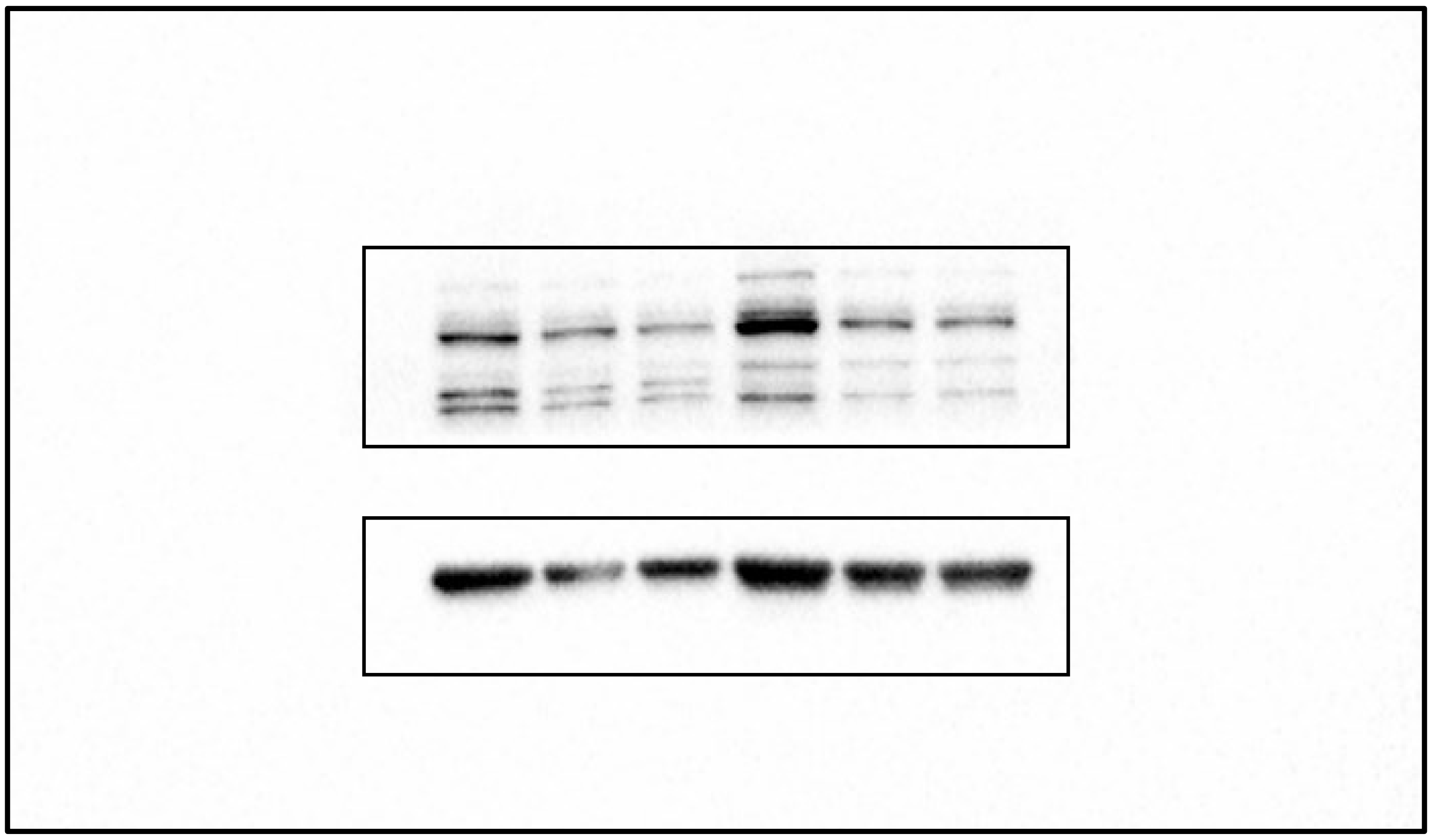

Fig 4. D

Nestin

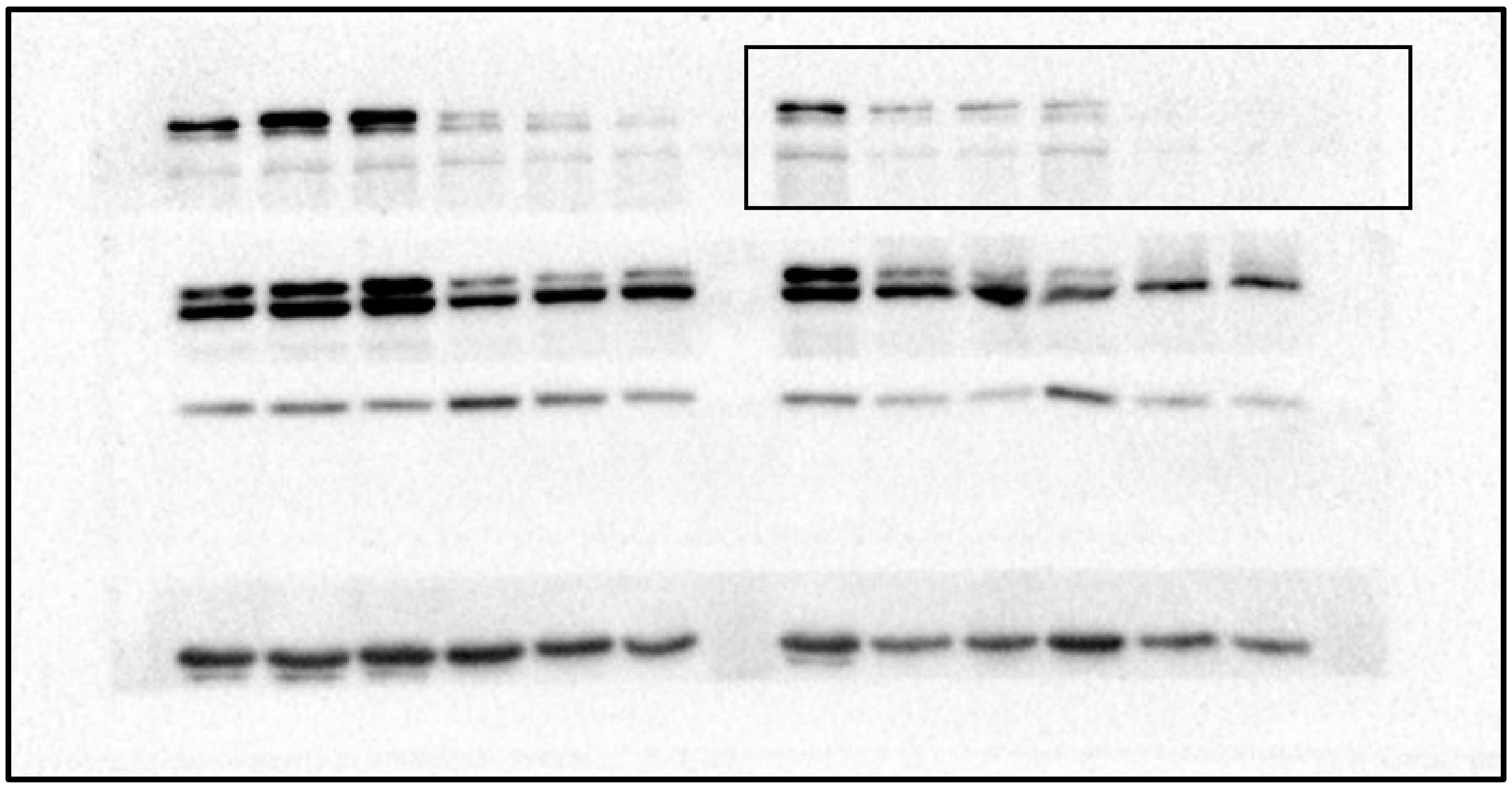

TCF7

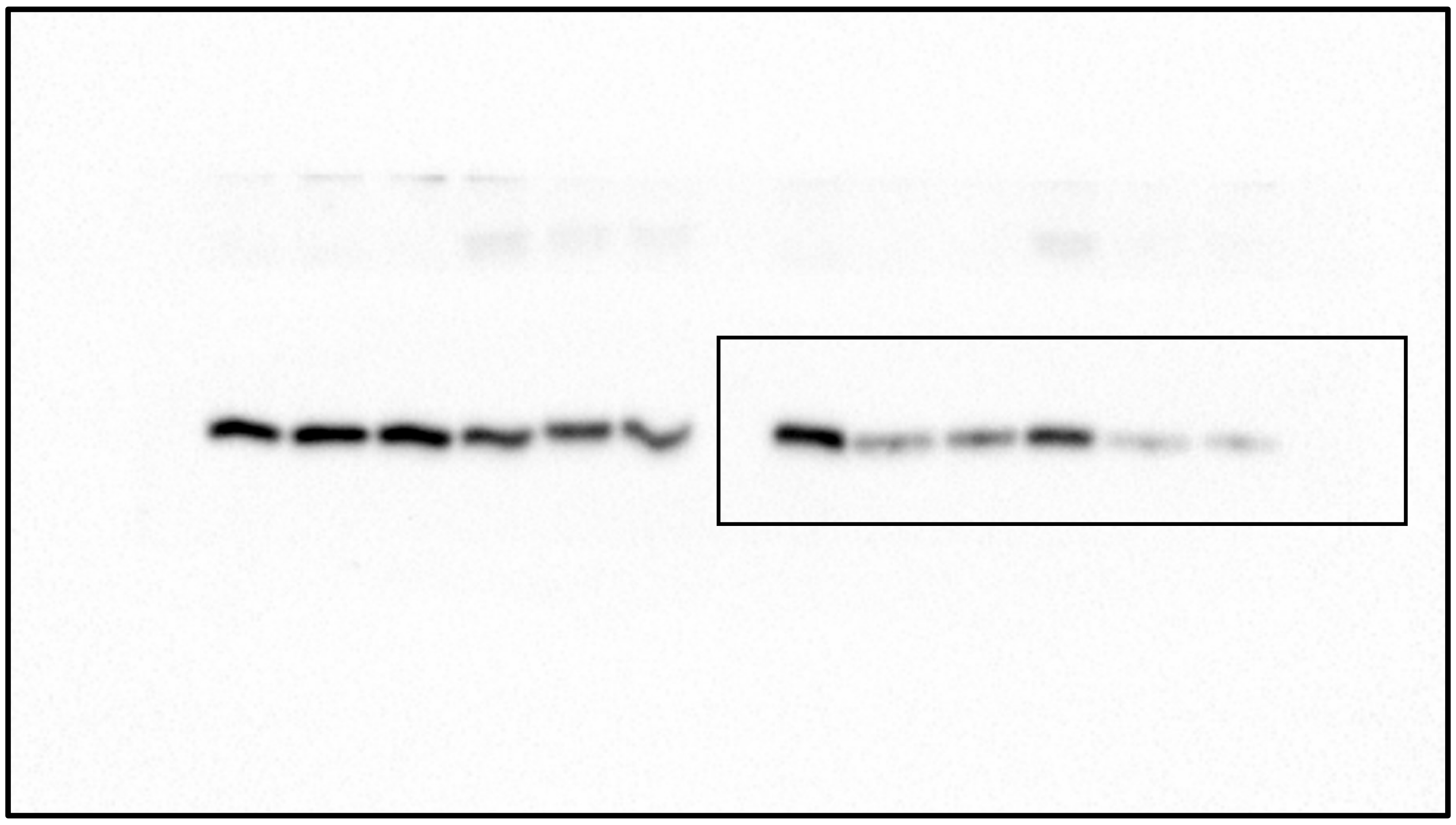

GAPDH

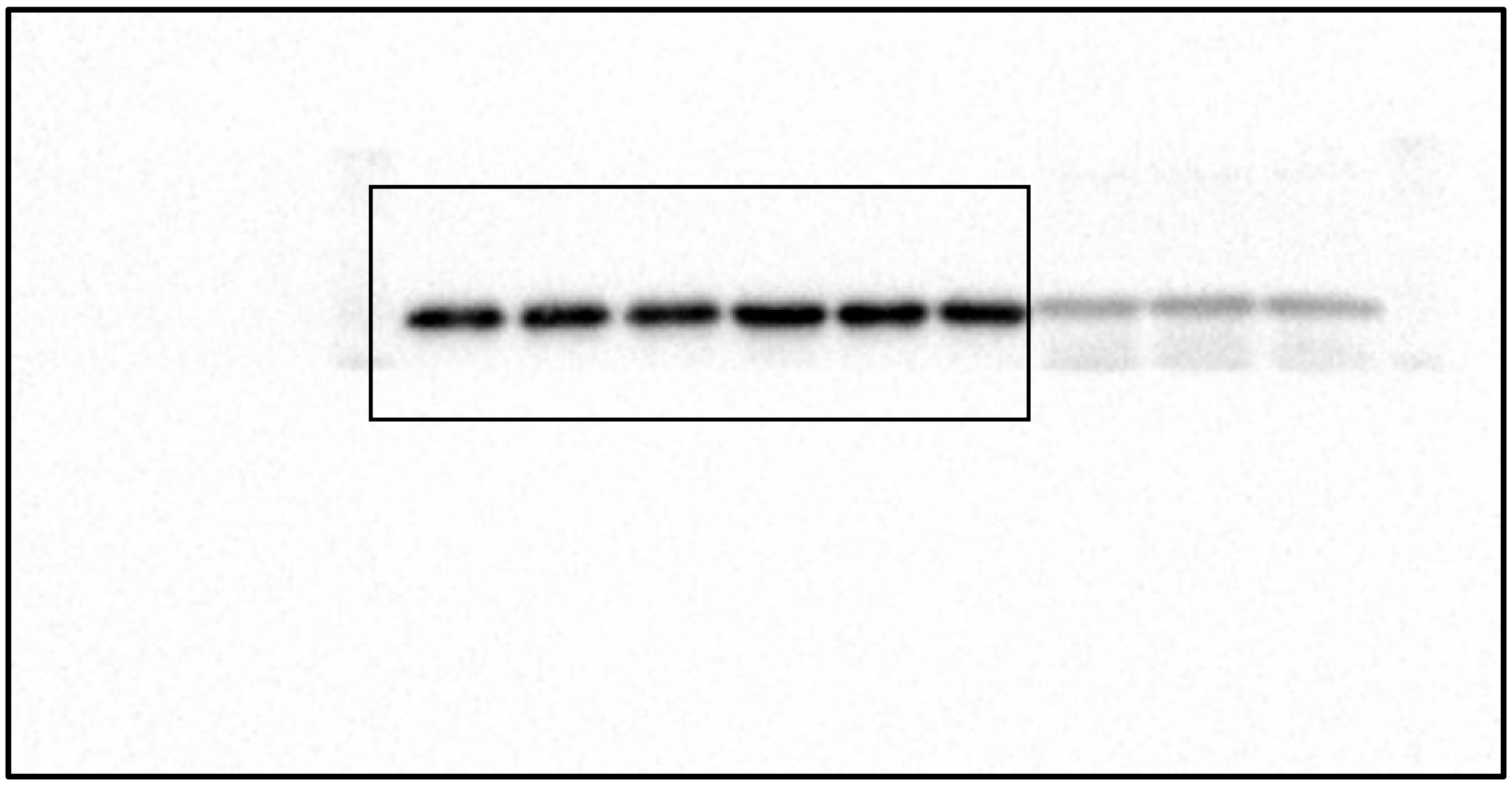

Supplement: Supplementary file 1 — Supplementary information [file 41598_2017_17340_MOESM1_ESM.pdf]
